# What Ministry of Health Bill Must Not Be

**Published:** 1918-11-09

**Authors:** 


The Workers' Newspaper of Administrative Medicine and Institutional Life, Administration, National Insurance and Health.

No. 1692, Vol. LXV. SATURDAY, NOVEMBER 9, 1918. PRICE TWOPENCE

### WHAT MINISTRY OF HEALTH BILL MUST NOT BE.

The public mind is getting restive in regard to the delays which have continuously occurred in formulating a scheme for a Ministry of Health, untrammelled and untied up by many administrative duties and details, conceived and built up upon an all-embracing plan firmly placed and resting on new ideas. This will call for the introduction of fresh blood to a controlling extent, which will secure to the country men of ability and independence, free from the trammels of tradition and the many entangling blots and impediments which at present are costing the people of these islands the infliction and continuance of evils which should become impossible under the wise administration of a Ministry of Health of the best conceivable type. We wish this article to avoid details and abstain from the introduction of points which have given rise to many existing differences representing various parties, each of which has some particular axe to grind, which may be frankly stated to belong for the most part to ideas and methods which the nation must see find no place in the new Act required to constitute the Ministry of Health which the welfare of the people and the national health imperatively demand. Lord Rhondda, as the *Times* has pointed out, believed that there were powerful subterranean influences at work to defeat the project he had in view for a Ministry of Health. The delays which have occurred, and the indications so far published of the trend and shape of such influences, being largely vested interests, manifest that they have proved mainly inimical to the preparation of a Bill to combine all the contending and rival interests involved, which Lord Rhondda, with his keen apprehension, so accurately described as subterranean influences calculated to defeat the project of a Ministry of Health of the only type which would meet adequately the nation's needs.

Lady Rhondda, in an interview published in the *Weekly Dispatch* of the 3rd instant, gives emphasis to her father's warning, and we venture to hope that the people throughout the country will be aroused to the keenest and most resolute opposition to any Bill for a Ministry of Health which does not provide for, as Lady Rhondda wisely declares, "just the health of the people," all-embracing in character, but definitely exclusive of the organised efforts to hamper and impede its usefulness, and of the largely vested interests behind the scenes which are struggling to be tacked on to the Bill which Dr. Addison, the Reconstruction Minister, has in preparation. The latest report is that the Bill at present drafted, so far as it has advanced, is to bear the title of "The Ministry of Health and Local Government Bill." This announcement has been generally accepted as indicating that the vested interests feared by Lord Rhondda have, by the pressure they have brought to bear upon the Minister of Reconstruction, succeeded in introducing clauses which will hamper the Bill and render it largely inefficient and unacceptable. Instead of being kept free from the exclusive element which hampers research, the old conventions that crush progress, and above all from vested interests which would try to check its workings, the Bill under the new title threatens to be largely dangerous to the nation by embracing some or all of these evils.

We give Dr. Addison credit for desiring to join all sections of opinion as far as the public interests permit. The people's interests are diametrically opposed to the inclusion in a Bill for setting up a Ministry of Health of vested interests which would try to check its workings and defeat its object. That is why the announcement that the Bill so far drafted, bearing the title of "The Ministry of Health and Local Government Bill," must be withdrawn, if the new Act is to mean just the health of the people, is to do the work so urgently required, and to protect the health and uphold and defend the life-giving properties, which all classes of the community will require from a Ministry of Health, to secure for them the blessings which freedom and democracy can and must give to every resident within the confines of the United Kingdom.

Lord Rhondda, we are confident, would have secured a Bill bearing the simple title of "The Ministry of Health Bill," which would have guaranteed to the people of these islands all that they needed, and must secure under any Act bearing this title and receiving the Royal assent. Lady Rhondda justly insists that public opinion where health is concerned is and must be in fact women's opinion. A Ministry of Health must realise this, or it will fail to win popular approval. Any Bill will invite certain defeat should the Minister of Reconstruction, for any reason, suffer men with other axes to grind than just the health of the people, to hamper, as it is credited that the scheme bearing the title of "The Ministry of Health and Local Government Bill" is hampered.

We hope Lady Rhondda, with her wise head and marked ability, will retain her father's place by bringing the late Lord Rhondda's points to the full knowledge and apprehension of the people, so that the present triumph of vested interests, which the announced title foreshadows, may be promptly defeated and eliminated.

